# Analysis of the Efficacy of Red Blood Cell Transfusion in Very Low Birth Weight Infants and Construction of a Prediction Model

**DOI:** 10.1007/s12288-026-02410-x

**Published:** 2026-04-04

**Authors:** Zulan Ouyang, Feng Zhao, Jianping Ni, Yanhong Li

**Affiliations:** 1 Department of Blood Transfusion,Women and Children’s Hospital of Ningbo University, Ningbo, 315012 Zhejiang China; 2 Neonatal Intensive Care Unit, Women and Children’s Hospital of Ningbo University, Ningbo, 315012 Zhejiang China

**Keywords:** Very low birth weight infants, Red blood cells, Transfusion efficacy, Prediction model

## Abstract

This study aimed to identify key determinants influencing red blood cell (RBC) transfusion efficacy in very low birth weight (VLBW) infants and develop a clinically applicable predictive model. We conducted a retrospective analysis of 993 eligible very low birth weight (VLBW) infants who received leukoreduced suspended RBC transfusions in the Neonatal Intensive Care Unit of Women and Children’s Hospital of Ningbo University between January 2020 and December 2023. Comprehensive data collection encompassed infant characteristics, clinical parameters, and donor information.Using logistic regression analysis, we identified five independent predictors for ineffective RBC transfusion: birth weight, postnatal age at transfusion, weight during transfusion, transfusion volume, and pre-transfusion hemoglobin levels. These variables were incorporated into a nomogram prediction model developed using R software. The model demonstrated strong predictive performance, with calibration curves showing excellent agreement between predicted and observed outcomes (AUC = 0.799). Our findings provide valuable insights into transfusion efficacy in VLBW infants and offer a practical tool for clinical decision-making.The validated nomogram enables individualized risk assessment, facilitating precision transfusion practices and potentially improving outcomes for this vulnerable patient population. This study represents a significant step toward optimizing transfusion strategies in neonatal care.

## Introduction

Very low birth weight (VLBW) infants, defined as infants with a birth weight < 1500 g [1], often require red blood cell (RBC) transfusions due to physiological anemia, iatrogenic blood loss, and other factors [2, 3]. These transfusions aim to improve anemia and enhance tissue oxygenation by replenishing the number of red blood cells in the body. In addition, the smaller the premature infant, the more likely they are to need earlier and more frequent transfusions [4–6]. However, there is also the situation of ineffective red blood cell transfusion, which affects the improvement of the child’s condition and leads to repeated transfusions. Accurate assessment of transfusion needs and prediction of efficacy are crucial for clinical decision-making. The influence of blood donor demographics and genetic characteristics, component processing, and recipient factors on transfusion efficacy is an important area of research in transfusion medicine [7, 8]. It is known that the inherent variability of the blood donor population affects the quality of blood components, but the impact on transfusion recipient outcomes is unclear. At present, most studies focus on the transfusion strategy and risk factor analysis of VLBW infants. This study focuses on the factors affecting the efficacy of red blood cell transfusion, including the condition of the child, red blood cell storage, and blood donor factors. This study aims to explore the key factors influencing the efficacy of red blood cell transfusion in VLBW infants through retrospective analysis, and to construct a prediction model to forecast the probability of transfusion effectiveness in VLBW infants. Early intervention targeting these influencing factors will be implemented to reduce the occurrence of ineffective transfusions, guide red blood cell transfusion in VLBW infants, and provide a basis for clinical individualized transfusion decision-making.

## Materials and Methods

### Study Design

**Fig. 1:**
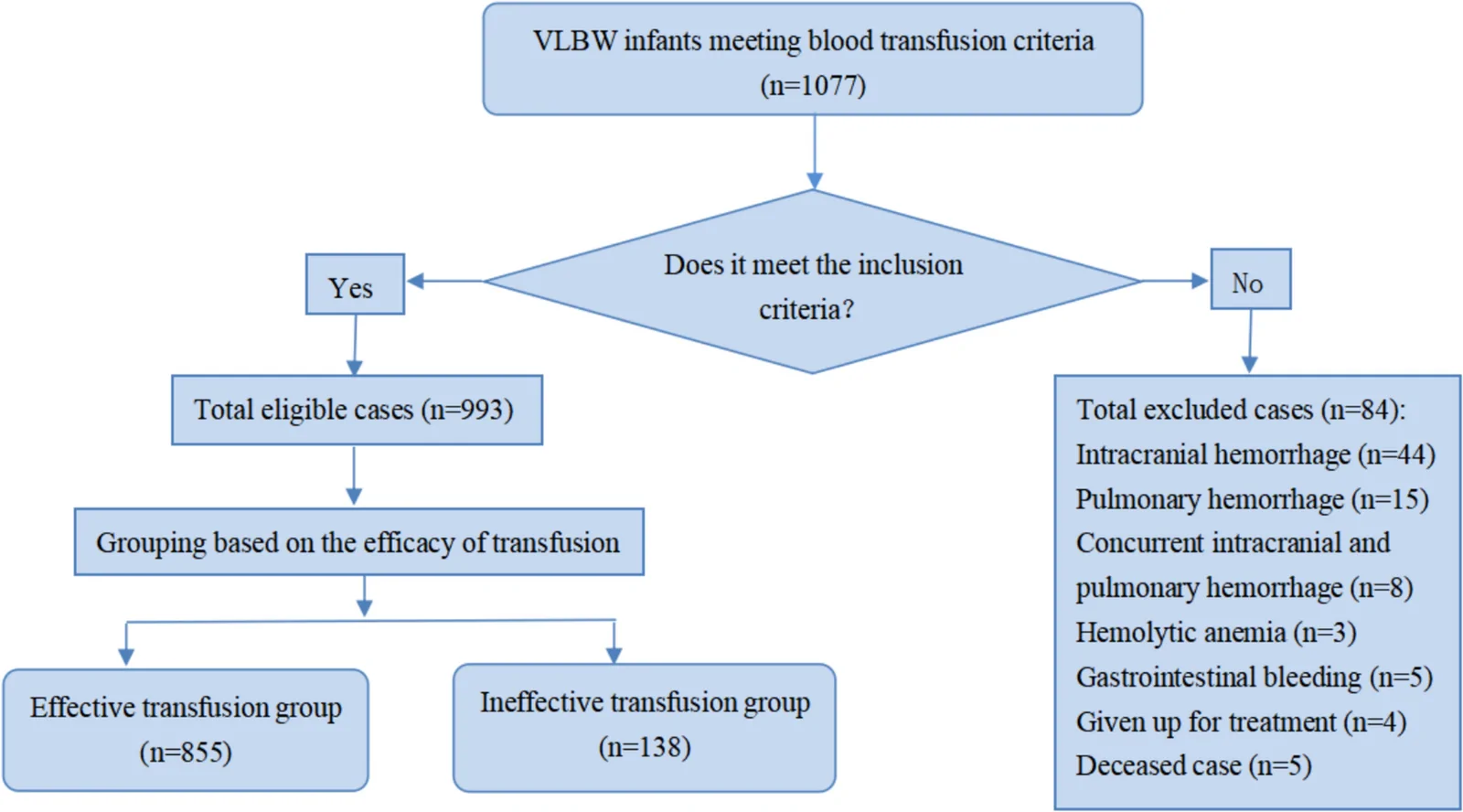
Flowchart of inclusion and exclusion criteria for study subjects

This is a single-center retrospective study initiated by the Women and Children’s Hospital of Ningbo University. From January 2020 to December 2023, 1077 cases of VLBW infants who received leukoreduced suspended RBC transfusions and met the transfusion criteria in the Neonatal Intensive Care Unit were enrolled. Excluding 84 cases, 993 infants met the inclusion criteria.They were divided into an effective group of 855 cases and an ineffective group of 138 cases based on the efficacy of transfusion, as shown in Fig. 1. The sample size was determined based on the complete clinical data availability of eligible VLBW infants over a 4-year period. Post-hoc power analysis confirmed that the final sample size of 993 infants provided sufficient statistical power (> 95%) to detect differences in transfusion efficacy (α = 0.05).Eligible participants met the following inclusion criteria: birth weight < 1500 g, anemia diagnosis meeting clinical transfusion thresholds, hospital admission within 24 h post-delivery, and minimum hospitalization duration of 7 days. We excluded infants with primary hematologic disorders (including hemolytic anemia, erythrocyte production defects, or other blood dyscrasias), significant confounding conditions (exchange transfusion, traumatic hemorrhage, occult blood loss, hemorrhagic disorders, or major congenital anomalies), or treatment withdrawal due to critical illness.

### Patient Data

We retrieve patient data through the hospital blood transfusion information system and the medical record information system. The collected data include: demographic characteristics, current clinical status, diseases, complications, and the results of blood routine tests conducted 24 h before and after blood transfusion.

### Donor Data

Donor information was traced through the product identification number of the suspended red blood cells transfused to the patients. All data were provided by the Ningbo Blood Center. The collected donor data include the following aspects: (1) Basic information: age, gender, lifestyle habits (including smoking, alcohol consumption, etc.); (2) Donation records: volume per donation, cumulative number of donations; (3) Blood product characteristics: blood collection date, storage time of the red blood cell product (days from collection to transfusion), and ABO blood type. The entire data collection process adhered to the standard operating procedures of the Ningbo Blood Center to ensure data completeness and accuracy. To ensure privacy protection, all donor information was anonymized, retaining only the characteristic parameters relevant to the study.

### Red Blood Cell Infusion in Suspension

The leukoreduced suspended red blood cell products administered in this study were all supplied by the Ningbo Blood Center in accordance with standard blood banking protocols. The red blood cells were stored at 4 ± 2 °C with CPDA-1 preservative solution, and the shelf life was 35 days. Transfusions were performed with ABO- and Rh(D)-identical blood that was crossmatch-compatible.The transfusion volume was 10–20 mL/kg, and the transfusion duration was 4 h.

### Indications for Blood Transfusion

The transfusion indications were established referring to the transfusion guidelines for preterm infants in *Practical Neonatology* (4th edition) [9], with the specific criteria as follows:(1) For moderate mechanical ventilation infants: Hemoglobin (Hb) ≤ 110 g/L or Hematocrit (HCT) ≤ 35%;(2) For mild mechanical ventilation infants: Hb ≤ 100 g/L or HCT ≤ 30%;(3) For infants requiring oxygen therapy but no mechanical ventilation (with clinical symptoms of anemia): Hb ≤ 80 g/L or HCT ≤ 25%;(4) For asymptomatic infants: Hb ≤ 70 g/L or HCT ≤ 20%. Note: Clinical symptoms of anemia include but are not limited to tachypnea, feeding difficulties, slow weight gain, tachycardia, or hemodynamic instability.

### Indicators of Blood Transfusion Effectiveness

With reference to pediatric transfusion guidelines, red blood cell transfusion for infants is generally administered at a standard dose of 10–15 mL/kg [10]. Effective red blood cell transfusion is defined as achieving the expected improvement indicators. The increase in hemoglobin (Hb) after transfusion is calculated based on Hb values within 24 h before and after transfusion. An increase of 2–3 g/dL is considered effective, while an increase of less than 2 g/dL is considered ineffective.

### Statistical Analysis

All analyses were performed using SPSS version 27.0 and R statistical software. Categorical variables are presented as frequencies (percentages) and were compared using the chi-square test. Continuous variables were assessed for normality using Shapiro–Wilk tests. Normally distributed data are expressed as mean ± standard deviation and compared using Student’s t-test, while non-normally distributed data are presented as median (interquartile range, *P*_25_, *P*_75_) and analyzed using the Wilcoxon rank-sum test.Multivariable logistic regression was employed to identify independent risk factors. For predictive modeling, we employed the following R statistical packages: the "rms" package was used to construct clinical prediction nomograms, the "calibrate" package facilitated calibration curve analysis to assess model accuracy, and the "pROC" package generated receiver operating characteristic (ROC) curves with corresponding area under the curve (AUC) calculations to evaluate discriminative performance. All statistical analyses were conducted using two-tailed tests with an alpha level of 0.05 for determining statistical significance, and all reported *p*-values represent exact values unless below the computational threshold of 0.001.

## Results

### Characteristics of the Patients

This study enrolled a total of 993 eligible VLBW infants. The cohort had a median gestational age of 29 weeks (IQR27-30) and median birth weight of 1170 g (IQR 1020–1300). The population comprised 539 male infants (54.3%) and 454 female infants (45.7%), with no significant gender-based differences observed in transfusion efficacy (*P* > 0.05). The overall transfusion effectiveness rate was 86.1% (855/993).Notably, many infants presented with multiple concurrent conditions.The main disease diagnoses focused on neonatal respiratory distress syndrome (NRDS), neonatal pneumonia, and bronchopulmonary dysplasia (BPD), accounting for 92.4%, 91.5%, and 83.8% respectively. The cohort demonstrated prolonged hospitalization with frequent transfusion requirements, exhibiting a median of 3 transfusions per infant (range 1–17), with the majority (67.3%) receiving 1–3 transfusions.

Significant differences (*P* < 0.05) were observed between the effective and ineffective transfusion groups for multiple parameters:gestational age, birth weight,postnatal age at transfusion, weight during transfusion, pre-transfusion and post-transfusion hemoglobin levels,volume, dose, frequency, and interval.No significant differences (*P* > 0.05) were found in: Apgar scores, related diseases, storage time, donor gender, age, blood type or whether the gender of the donor and recipient match.Complete comparative data are presented in Table 1.

**Table 1:** Patient, blood donor, and RBC transfusion characteristics

Characteristic	Effective group (*N* = 855)	Ineffective group (*N* = 138)	*χ*^*2*^* /t/Z*	*P* value
Patient characteristics				
Sex*				
Men	465(54.4%)	74(53.6%)	0.028	0.867
Women	390(45.6%)	64(46.4%)		
Gestational age:median ( *P*_25_,*P*_75_), weeks**	29(28,30)	28(27,29)	-3.156	< 0.001
Birth weight, grams**	1180(1030,1300)	1110(950,1250)	-3.268	0.001
1-min Apgar score**	8(7,8)	8(6,8)	-1.632	0.103
5-min Apgar score**	9(9,9)	9(8,9)	-1.522	0.128
Postnatal age at transfusion,d**	25(15,40)	18(8,30)	-5.349	< 0.001
Weight during blood transfusion,g**	1670(1330,2010)	1430(1140,1820)	-4.715	< 0.001
Blood transfusion volume,ml**	30(24,36)	24(18,30)	-6.478	< 0.001
Blood transfusion dose,ml/kg**	18.75(15.75,19.59)	15.90(14.82,18.23)	-6.164	< 0.001
Hb before transfusion: Mean ± SD, g/dL***	10.05 ± 1.076	11.02 ± 1.147	9.750	< 0.001
HCT before transfusion, %***	29.93 ± 3.46	32.87 ± 3.78	8.036	< 0.001
Hb after transfusion, g/dL***	14.24 ± 1.67	12.19 ± 1.27	-13.81	< 0.001
HCT after transfusion, %***	41.72 ± 5.49	36.04 ± 3.80	-13.216	< 0.001
Hb increment,g/dL**	3.9(3.0,5.1)	1.4(0.9,1.7)	-18.874	< 0.001
Number of RBC transfusion episodes**	2(1,4)	2(1,3)	-2.524	0.012
Blood transfusion interval time,d**	7(0,14)	2(0,7)	-4.902	< 0.001
Diagnosis*				
Pneumonia of newborn	779(91.20%)	130(94.20%)	1.356	0.244
NRDS	792(92.6%)	126(91.3%)	0.300	0.584
BPD	718(84.0%)	114(82.6%)	0.164	0.686
Sepsis	315(36.90%)	56(40.90%)	0.767	0.381
Abnormal coagulation function	289(33.8%)	62(44.9%)	6.437	0.011
Hyperbilirubinemia of newborn	166(19.40%)	22(15.90%)	0.934	0.334
RBC storage age,d**	22.8(17,26.9)	22(17.5,27.19)	-0.320	0.749
Blood donors characteristics				
Age:median ( *P*_25_, *P*_75_), years**	38(23,47)	38(23,47)	-0.91	0.363

Characteristic	Effective group (*N* = 855)	Ineffective group (*N* = 138)	*χ2 /t/Z*	*P* value
Sex*				
Men	551(64.4%)	81(58.9%)	1.108	0.292
Women	304(35.5%)	57(41.1%)		
ABO status *				
A	235(27.5%)	45(33.0%)	1.379	0.711
B	300(35.1%)	47(34.0%)		
O	225(26.3%)	33(23.7%)		
AB	95(11.1%)	13(9.4%)		
Smoke*	226(26.5%)	26(18.8%)	2.469	0.116
Drink*				
No	290(34.0%)	51(37.0%)	0.635	0.728
Occasionally	282(33.0%)	40(29.0%)		
Frequently	277(32.4%)	47(34.0%)		
Blood donation volume,ml**	300(300,300)	300(300,300)	-0.235	0.814
Number of blood donations**	1(1,7)	1(1,8)	-0.298	0.765
Donor-recipient gender consistency*				
Yes	405(47.4%)	68(49.5%)	0.142	0.706
No	450(52.6%)	70(50.5%)		

Note *Categorical data were presented as numbers or percentages and compared by chi-square test.

**Data were presented as median(interquartile range, *P*_25_, *P*_75_) and compared by Wilcoxon rank-sum test.

***Data were presented as mean ± SD and compared by Student’s t-test.

Hb:hemoglobin;HCT: hematocrit.

### Independent Predictors of Transfusion Efficacy

We initially conducted univariate logistic regression analysis of all potential influencing factors (Table 2), selecting variables with *P* < 0.1 for subsequent stepwise regression analysis to identify key determinants of transfusion efficacy. Ultimately, independent predictive factors were determined using a multivariate logistic regression model (Table 3). The results showed that red blood cell storage time and donor-related factors (age, gender, number of blood donations) were not significantly associated with transfusion efficacy. In contrast, patient-related variables were significantly correlated with the outcomes of the multivariate model, including birth weight, postnatal age at transfusion, body weight at transfusion, transfusion volume, and pre-transfusion hemoglobin.

**Table 2:** Univariate logistic regression analysis on the Therapeutic effect of blood transfusion in VLBW infants

Characteristic	OR (95% CI)	*P*
Patient characteristics		
Gestational age, weeks	1.177(1.060,1.307)	0.002
Birth weight, g	1.002(1.001,1.003)	< 0.001
Weight during blood transfusion, g	1.001(1.001,1.001)	< 0.001
Postnatal age at transfusion,d	1.029(1.017,1.041)	< 0.001
Blood transfusion interval,d	1.020(1.007,1.034)	0.002
Blood transfusion volume,ml	1.073(1.050,1.097)	< 0.001
Hb before transfusion, g/dL	0.432(0.359,0.521)	< 0.001
Blood transfusion dose,ml/kg	1.235(1.148,1.329)	< 0.001
Number of RBC transfusion episodes	1.085(0.991,1.188)	0.077
RBC storage age,d	0.994(0.964,1.025)	0.707
Blood donors characteristics		
Age, years	1.009(0.988,1.029)	0.409
Sex	0.789(0.507,1.227)	0.293
Number of blood donations	0.987(0.919,1.060)	0.719

**Table 3:** Multivariate logistic Regression Analysis on the Therapeutic Effect of blood Transfusion in VLBW infants

Characteristic	OR (95% CI)	*P*
Birth weight, g	1.003(1.002,1.005)	< 0.001
Postnatal age at transfusion,d	1.025(1.003,1.047)	0.025
Weight during blood transfusion,g	0.997(0.996,0.998)	< 0.001
Blood transfusion volume,ml	1.140(1.086,1.196)	< 0.001
Hb before transfusion, g/dL	0.403(0.323,0.504)	< 0.001

### Prognostic Nomogram of the Probability of Effective Red Blood Cell Transfusion

**Fig. 2:**
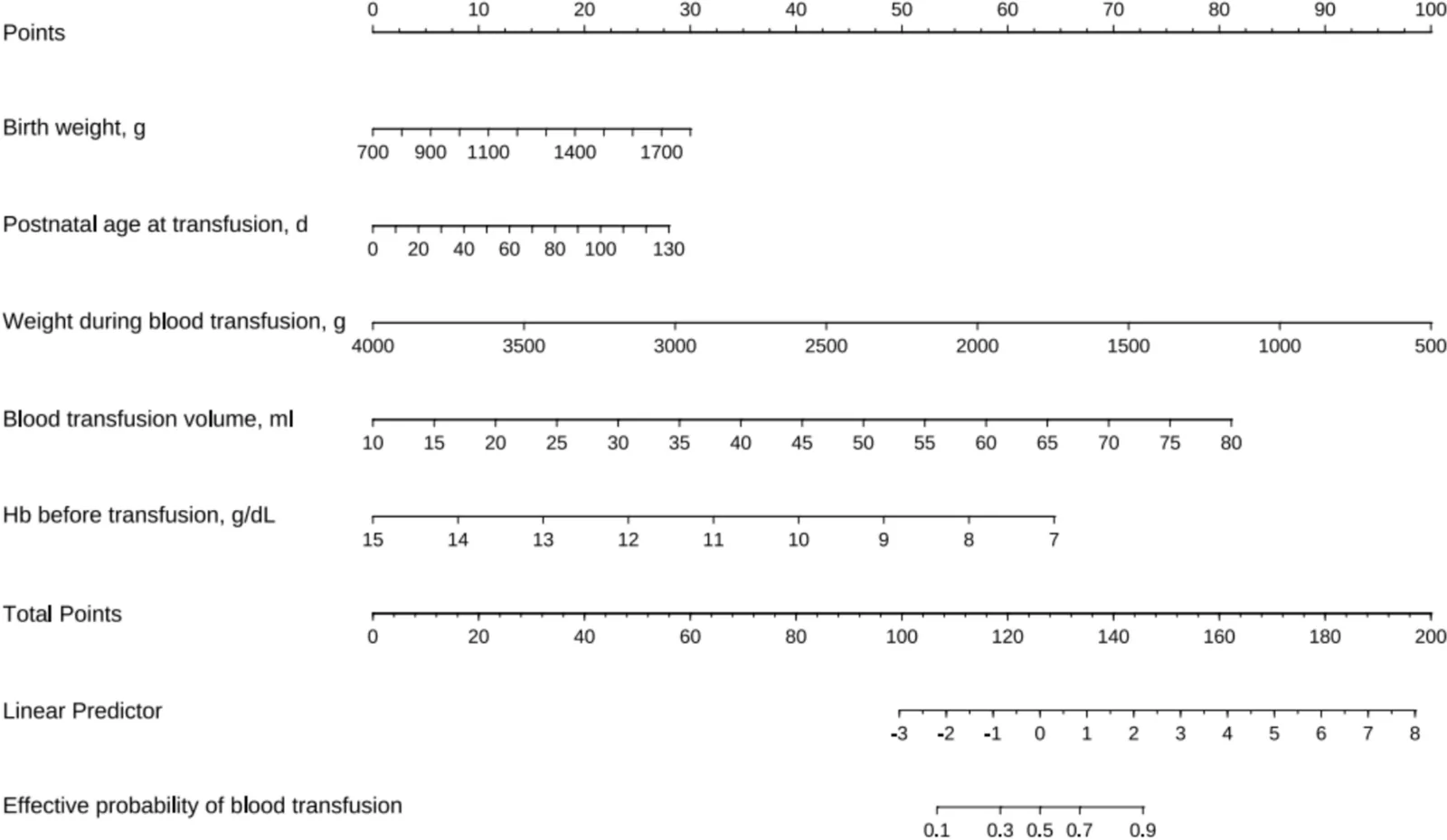
Nomogram for predicting red blood cell transfusion efficacy in VLBW infants

**Fig. 3:**
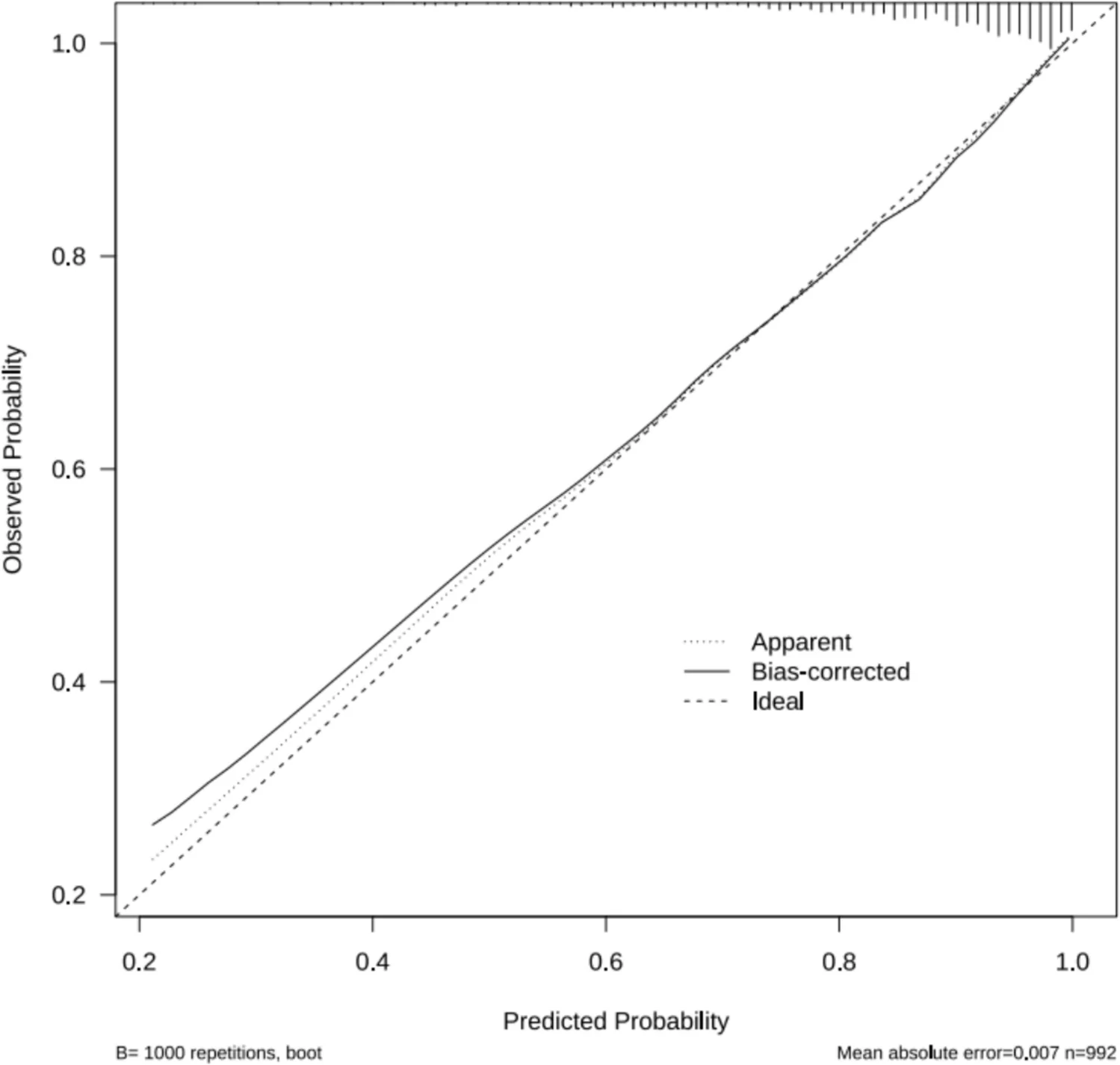
Nomogram correction curve. The predicted probability of the model is displayed on the x-axis, and the actual probability is displayed on the y-axis

Based on the results of multivariate analysis, we constructed a nomogram model (Fig. 2) to predict the efficacy of red blood cell transfusion. The model integrates key predictors, including 5 independent risk factors. A point scoring system was used to quantify the contribution of each factor. Each predictor corresponds to a points score through a vertical line, and the scores of each item are summed to obtain the total score. A higher score indicates a higher probability of effective transfusion. The calibration plot of effective transfusion probability shows excellent consistency between the nomogram prediction and the actual observation results (Fig. 3).

### ROC for Predicting RBC Transfusion Efficacy

**Fig. 4:**
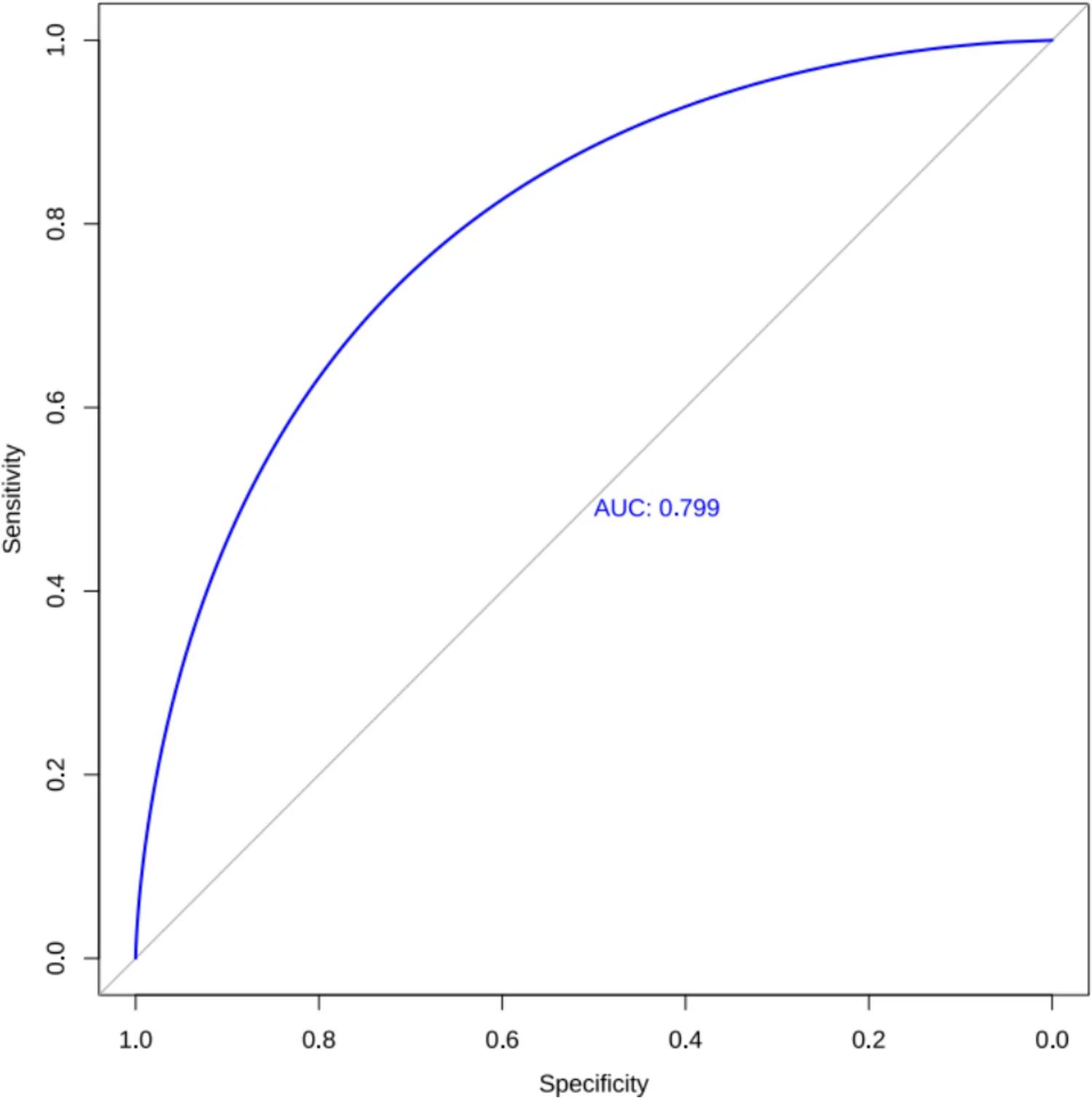
The characteristic curve of the receptor operator for predicting the therapeutic effect of red blood cell transfusion. AUC refers to the area under the receiver operating characteristic curve

The predictive efficacy analysis demonstrated robust performance of our transfusion outcome model, with the receiver operating characteristic (ROC) curve analysis yielding an area under the curve (AUC) of 0.799, as illustrated in Fig. 4.

## Discussion

Anemia in preterm infants represents a complex pathophysiological condition resulting from multiple contributing factors, including diminished erythropoietin (EPO) production, accelerated erythrocyte destruction, and impaired bone marrow function [11–13]. The clinical necessity for transfusion is particularly prevalent in this vulnerable population, with epidemiological studies demonstrating transfusion requirements in approximately 40% of low birth weight infants and up to 90% of VLBW infants [14]. Red blood cell transfusions confer significant physiological benefits by enhancing oxygen delivery through multiple mechanisms: elevation of circulating hemoglobin concentration, improvement in tissue oxygenation efficiency, and reduction in cardiac workload. While post-transfusion hemoglobin recovery serves as a conventional biomarker for evaluating transfusion efficacy and monitoring hematological improvement, clinical outcomes demonstrate considerable variability across recipients. The heterogeneous response to transfusion stems from a complex interplay of immunological and non-immunological mechanisms that may compromise transfusion effectiveness. Furthermore, substantial interindividual variation in transfusion outcomes arises from the tripartite interaction of donor-specific factors (including donor characteristics and blood component properties), transfusion product variables (such as storage duration and processing methods), and recipient-related determinants (encompassing clinical status and biological individuality) [15]. Notably, our findings emphasize that recipient physiological parameters exhibit the strongest association with transfusion outcome variability, underscoring the importance of patient-specific considerations in transfusion decision-making.In our cohort, approximately 14% of cases showed transfusion inefficiency, leading to repeated transfusions. The theoretical fates of red blood cells after transfusion into the body include normal metabolism, hemorrhagic loss, redistribution in the body (such as hepatic-spleen sequestration), infusion dilution, physicochemical destruction, and immune destruction.

This study retrospectively analyzed and identified key factors affecting the efficacy of red blood cell transfusion in VLBW infants, including gestational age, birth weight, weight at transfusion, transfusion volume, pre-transfusion Hb level, transfusion timing, and frequency.Univariate logistic regression analysis showed that low gestational age, low birth weight, low postnatal age, and small transfusion volume were significantly associated with poor transfusion outcomes. Specifically, infants with lower birth weights showed weakened therapeutic responses to transfusion. This may be attributed to two key factors: (1) The absolute transfusion dose given to smaller infants is relatively low, which may limit clinical efficacy; (2) The physiological functions and metabolic capacity of low birth weight newborns are immature.

This study shows that hemoglobin before red blood cell transfusion has a significant impact on the transfusion effect. The lower the hemoglobin before transfusion, the more red blood cells may be transfused, and thus the more hemoglobin increases. This is consistent with the conclusion of Roubinian NH’s [16]study, which found that the hemoglobin level before transfusion is a significant predictor of hemoglobin increase. Statistical analysis found no significant correlation between the disease of children and transfusion efficacy.

Our study revealed no impact of donor characteristics or red blood cell storage duration on transfusion efficacy, contradicting DeSimone RA’s [17] report that donor gender, age, and hemoglobin levels correlate with efficacy of red blood cell transfusion in VLBWI. This discrepancy may stem from methodological differences: their study analyzed the relationship between donor gender/age and hemoglobin increment, whereas we investigated whether these factors affect transfusion efficacy. Saqlain N [18] also found no significant association between donor gender and hemoglobin increment, consistent with our results.Meanwhile, this study also found no impact of gender mismatch between the recipient and donor on the efficacy of blood transfusion. Although current donor factors analyzed showed no effect, we cannot confirm whether other factors (e.g., donor hematocrit, pregnancy history and medication use) influence transfusion efficacy, warranting further investigation. Additionally, no significant difference in efficacy was found across different donor blood types. A meta-analysis of 14 published RCTs including 26,374 pediatric and adult patients randomized to standard vs. short-stored blood found no benefit of the latter [19], consistent with our findings. Notably, our study focused only on hemoglobin increment within 24 h post-transfusion, without exploring long-term efficacy.

Nomograms are widely used in medical research to integrate clinical features and visualize regression analysis, revealing how different factors impact outcomes [20–22]. In the modal diagram model, all factors influence the infusion effect. It is not that the more blood transfused, the better the outcome; rather, the model provides a quantitative assessment of individualized transfusion efficacy. Current studies on red blood cell transfusion efficacy mainly analyze influencing factors, lacking predictive models.Independent predictors were identified through logistic regression, and a nomogram model was constructed using R software to quantitatively calculate the probability of effective blood transfusion, assisting clinicians in active intervention [23].This model achieved an AUC of 0.799, and its internal validation calibration curve closely matched the actual curve, demonstrating good diagnostic value. This model provides a quantitative tool for evaluating transfusion efficacy in VLBW infants, potentially promoting precision transfusion practices. The model, by integrating multiple factors, can help identify infants with potentially poor efficacy after blood transfusion, and adjust treatment plans in advance (such as optimizing transfusion thresholds and combining EPO) [24]. The nomogram’s visual interface facilitates bedside rapid assessment.

While this study provides valuable insights into transfusion efficacy predictors in VLBW infants, several important limitations should be acknowledged: (1) The retrospective design may introduce selection bias and confounding factors, including variability in clinical transfusion indications and unmeasured maternal factors such as gestational anemia; (2) The single-center nature limits generalizability, as institutional practices may differ; (3) The hematocrit (HCT) data of blood donors was not routinely recorded in the archives reviewed in this study. The absence of this parameter may affect the comprehensive evaluation of the correlation between blood donors’ blood characteristics and transfusion outcomes. Future research directions should focus on: (1) Establishing collaborative multicenter studies to validate and refine the predictive model; (2) Implementing prospective study designs with standardized protocols to minimize bias; (3) Incorporating additional clinical, laboratory and blood donor parameters to enhance model precision. These efforts will be crucial for developing robust clinical decision tools that can optimize transfusion strategies and improve outcomes in this vulnerable population.

In conclusion, this study identified factors influencing red blood cell transfusion efficacy in VLBW infants and constructed a clinically applicable predictive model. Prospective studies are required to optimize the model for individualized therapy and improved outcomes.
